# An observational analysis of risk factors associated with symptomatic third molar teeth

**DOI:** 10.12688/wellcomeopenres.17673.2

**Published:** 2023-07-17

**Authors:** Douglas Bruce, Tom Dudding, Mark Gormley, Rebecca C Richmond, Simon Haworth

**Affiliations:** 1 Bristol Dental School, University of Bristol, Bristol, UK; 2 The MRC Integrative Epidemiology Unit at the University of Bristol, University of Bristol, Bristol, UK

**Keywords:** Third molars, wisdom teeth, pericoronitis, risk factors, genetics, ALSPAC

## Abstract

Background: Third molar teeth (wisdom teeth) are a common cause of pain and infection in young adults. The study aimed to describe the prevalence of symptomatic third molar teeth and identify factors which predispose to third molar symptoms in a birth cohort.

Methods: An observational study was undertaken nested in the Avon Longitudinal Study of Parents and Children (ALSPAC), a birth cohort based in south west England. The main outcomes were self-reported third molar pain, swelling and treatment for third molar problems, taken from questionnaires completed at age 23 years. The exposures including sex, dental history, socioeconomic status, diet, and genetic factors were obtained from earlier ALSPAC data.

Results: In total 4,222 ALSPAC participants responded to one or more questions about third molar teeth. The final sample included more female participants than male participants. The majority of participants (56.6%) reported at least one episode of pain associated with their third molars. Females had greater odds than males of reporting swelling (adjusted odds ratio (OR) 1.97; 95%confidence interval (CI) 1.56, 2.51), pain (adjusted OR=1.96; 95%CI 1.56, 2.51) and receiving both non-surgical and surgical treatment (adjusted OR=2.30; 95%CI 1.62, 3.35, adjusted OR=1.54; 95%CI 1.17, 2.06 respectively). Participants with previously filled teeth had greater odds of third molar extraction. There were no strong associations between index of multiple deprivation (IMD) score or sugar intake and the third molar outcomes. There was weak evidence for a genetic contribution to third molar pain.

Conclusions: Symptomatic third molars are common in this age group, with over half of the participants reporting pain or other symptoms. Female participants had greater odds for third molar pain, swelling and treatment.

## Introduction

Third molars, also known as wisdom teeth, are usually the last teeth to develop in humans, erupting in the late teenage years to early twenties ^1^. These teeth are often developmentally absent ^2^ or unfavorably positioned ^3^, and have highly variable crown and root morphology ^4^. Third molar impaction is a common problem ^5^, which can result in communication between the residual follicle space and the oral cavity, leading to bacterial ingress and infection ^6, 7^.

The management of impacted third molars remains an area of ongoing debate. In the United Kingdom, the National Institute of Health and Care Excellence (NICE) published guidance in 2000, suggesting third molar teeth should only be removed under specific clinical situations, discontinuing the practice of prophylactic removal ^8^. Two decades later, there is a growing body of evidence which challenges this guidance. While prophylactic surgery has problems, there are also risks associated with leaving third molars *in situ*, including pericoronitis, caries, periodontitis, and cyst development ^9– 14^ . A Cochrane review by Ghaeminia *et al.* concluded there was insufficient evidence to determine whether asymptomatic, disease‐free impacted third molars should be removed or retained ^15^, and clinicians need to weigh up the risks and benefits of different management approaches on a case by case basis in discussion with the patient. There is also a consideration to be made about the impact of third molar surgery on patients in terms of the impact to adjacent teeth and structures. Long term retention of mesially impacted third molars may result in caries on the lower second molar whilst surgical removal may result in periodontal damage at the distal aspect of the second molar ^16^. Alongside changes in guidance for the assessment of third molars, the management of post-operative complications through surgical technique, pharmaceuticals and more recently phytotherapy have been investigated ^17^. At the present time, common management strategies for mandibular third molar teeth range from clinical review and surveillance, to extraction of opposing maxillary third molar, coronectomy and surgical removal ^6^.

Ideally, a shared decision making process about treatment options would involve accurate assessment of risk factors for third molar pathology in addition to discussion of patient symptoms and preferences. People with multiple risk factors may benefit from early surgical management, as the complexity of third molar removal increases with age ^18^, while those with fewer risk factors might benefit from a period of active surveillance and conservative management. This would enable resources to be directed towards patients who are most likely to require future surgical management ^19^, while avoiding surgery for patients who are unlikely to develop problems.

There is, therefore, a need to understand the risk factors for developing third molar problems. Both host and environmental factors affect other dental diseases such as periodontal disease and caries ^20, 21^, but there is relatively little evidence to show which risk factors are associated with third molar pathology. The study aimed to describe the prevalence of symptomatic third molar teeth and associated treatment and to also identify factors which predispose to third molar symptoms in a birth cohort.

## Methods

### ALSPAC cohort

The Avon Longitudinal Study of Parents and Children (ALSPAC) is a large population-based birth cohort study ^22^. Pregnant women living in the former county of Avon (South West England, UK) with expected dates of delivery between 1 ^st^ April 1991 and 31 ^st^ December 1992 were invited to take part in the study. A total of 14,541 pregnancies were enrolled in the study, resulting in 14,062 live births and 13,988 children who were alive at 1 year of age. When these children were approximately 7 years of age, an attempt was made to enlarge the ALSPAC study by recruiting additional eligible people who had failed to join the study originally. A total of 913 additional children were enrolled though these efforts. This means the total initial sample size for the present study (with outcomes after the age of 7 years) is 15,454 pregnancies resulting in 14,901 children alive at 1 year of age. The ALSPAC study is ongoing, and the indexed children are now adults, many with children of their own. The study recruitment and design has been described in detail previously ^22– 24^. Study data was collected and managed using REDCap electronic data capture tools hosted at the University of Bristol. REDCap (Research Electronic Data Capture) is a secure, web-based software platform designed to support data capture for research studies ^25^. Comprehensive phenotype, genetic and environmental information has been collected from both mothers, their partners and offspring at multiple time points. Ethical approval for the ALSPAC study was obtained from the ALSPAC Ethics and Law Committee. Informed consent for the use of data collected via questionnaires and clinics was obtained from participants following the recommendations of the ALSPAC Ethics and Law Committee at the time. Consent for biological samples has been collected in accordance with the Human Tissue Act (2004). Approval for the analysis reported in this article was obtained from the ALSPAC study executive (project reference B3482). Participants were eligible for inclusion in the present analysis if they submitted a valid response to one or more questions about third molar problems (see below).

### Data collection

The host and environmental factors examined for this study were measured at numerous time points throughout the study. Some questionnaires were completed by the participant’s parents and others by the participant themself.

Sex was recorded by the midwife at the time of birth and recorded as either male or female. IMD scores were assigned according to a participant’s home post code at age 13.8 years when they completed the *Travelling Leisure and School* questionnaire. To minimize disclosure risks, the scores were divided into quintiles 1 to 5, with 1 being the least deprived and 5 the most deprived, and the quintiles were used as an ordered categorial variable in subsequent analysis.

Sugar intake was measured at the *Teen Focus 2* research clinics which were open to all members of the ALSPAC cohort from ages 12.5 years to 15.2 years. The mean age of participants at these clinics was 13.8 years. Sugar intake was reported via means of three-day dietary diaries and coded as a continuous variable with the units being grams of sugar per day.

Dental anxiety, and record of previous extractions were measured at the *Teen Focus 4: Focus at 17* research clinic. The age range was 16.25 years to 20 years with the mean age being 17.8 years. The questions regarding dental anxiety mirrored those used in the Corah anxiety scale, with 4 question stems asking how the participant would feel in different situations ( **Provided in full in extended data**). Each question stem had multiple responses, where lower scores indicate low levels of anxiety. Two question stems included options for reporting they had never received dental treatment. If participants selected this option (692 individuals) their response for these two question stems was replaced with the median response.

Participants at the *Teen Focus 4* clinic were asked how many teeth they have had taken out because they were ‘bad’, which was used as a proxy for previous treated dental caries experience. This variable was coded as a categorical variable with 3 levels (none, 1–4 and more than 4 previous extractions).

Data regarding the outcomes relating to third molar pathology and treatment were collected in the *Me @ 23* questionnaire which was completed sent to participants at age 23 years. Participants were asked if they had had pain or swelling from their wisdom teeth and, if so, how many episodes they had (1, 2–3, 3–4 or 5 or more times). They were also asked if they had any wisdom teeth removed or any other treatment to wisdom teeth when they were causing pain. Responses to these questions were summarized as binary variables of symptoms or no reported symptoms, and treatment or no reported treatment. The *Me@23* questionnaire also asked when the last time the young person went to the dentist. This was used as a proxy for dental attendance frequency, reported as either irregular (greater than two years between appointments) or regular (less than two years between appointments). They were also asked how many of their teeth had fillings or other restorative treatment such as crowns. This was treated as a proxy for previous caries experience and was coded as a categorical variable with 4 levels (no, 1–4, 5–9 and more than 9 teeth filled).

The questions asked in the ALSPAC surveys can be found in the extended data.

Please note that the study website contains details of all the data that is available through a fully searchable data dictionary and variable search tool ^26^. Data were retrieved in August 2020.

### Statistical analysis

Continuous variables were described using means and standard deviation. Categorical variables were described as counts and percentages. Logistic regression analysis was conducted using the *glm()* function to investigate the association between host and environmental exposures and self-reported outcomes. Logistic regression included unadjusted models, and models adjusted for age, sex and IMD Score. Analysis was performed using R (version 4.0.2).

### Genetic susceptibility

The variation in each outcome attributable to common single nucleotide polymorphisms (SNPs) was estimated with genetic restricted maximum likelihood analysis (GREML) using Genome-wide Complex Trait Analysis (GCTA) ^27, 28^. This analysis tests whether people who are more genetically similar are also more phenotypically similar, to infer whether genetic factors influence a trait.

DNA was extracted at various time points during the study from either blood, cell line or mouthwash samples and there were multiple builds of the genetic data. The genotype data used for this analysis was originally generated in collaboration between the Wellcome Trust Sanger Institute in the UK and the Laboratory Corporation of America using the Illumina HumanHap550 genotyping platforms. Quality control filtering was done with the PLINK (v1.07) software. SNPs with a minor allele frequency of < 1%, call rate < 95% and Hardy-Weinberg equilibrium (HWE) P < 5x10 ^-7^ were removed. The initial data included 9,912 individuals with 609,203 SNPs. Those with extreme or undetermined autosomal heterozygosity, those with insufficient sample replication (0.1) and >3% missingness have been removed leaving 9,115 individuals and 500,527 SNPs ^29^. ALPSAC children were phased using ShapeIt V2 to phase the Haplotype Reference Consortium (HRC) panel (39,235,157 SNPs) ^30^. Genotype imputation was performed with the Michigan Imputation Server using the Haplotype Reference Consortium (HRCr1.1) panel.

A subset of common genetic variants (minor allele frequency of 0.05 or greater) was then used to construct a genetic relatedness matrix and participants related at the first-degree level or closer (identity by state 0.125 or greater) were excluded. The final sample size with non-missing phenotypic data included 2,771 participants. Variation in the outcomes attributable to genetic factors was expressed as a proportion of the total phenotypic variance.

## Results

**Figure 1.**
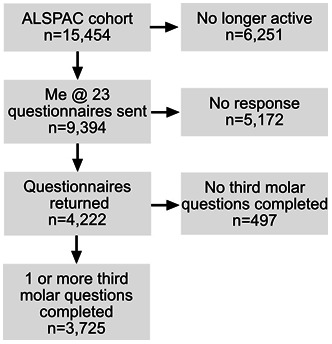
Flow chart of the final study sample.

A total of 9,394 participants were sent the ‘Me @ 23’ questionnaire. Participants who submitted a valid answer to at least one of the four questions related to third molar symptoms were included in the study ( Figure 1.).

The final study sample contained more female participants than male (66% female) and were predominantly from a less deprived background (the most common IMD quintile score was 1 with 30% of participants being from this quintile). Most participants reported attending a dentist regularly (82%) ( Table 1).

**Table 1.** Descriptive characteristics of the study population (SD: standard deviation).

	Reported third molar symptoms or treatment Total=3725
**Sex (%)** Male Female Missing	1,257 (33.7) 2,455 (66.2) 13 (0.002)
**Index of multiple deprivation (IMD) (%)** 1 2 3 4 5 Missing	1,126 (30.2) 760 (20.4) 507 (13.6) 349 (9.4) 185 (5.0) 798 (21.4)
**Dental attendance (%)** Regular attender Irregular or non-attender Missing	3,048 (81.8) 636 (17.1) 41 (1.1)
**Dental anxiety** (COHRA scale) (%) <13 13 or above **Missing**	1,356 (36.4) 219 (5.9) 2,150 (57.7)
**Filled Teeth (%)** 0 1 – 4 5 – 9 >9 **Missing**	1,711 (45.9) 1,615 (43.4) 312 (8.4) 87 (2.3) 0 (0)
**Teeth extracted due to decay (%)** 0 1 –4 >4 Missing	1,336 (35.9) 149 (4.0) 90 (2.4) 2,150 (57.7)
**Diet** Mean daily sugar intake in Gram (SD) (n=1432)	115g (49g)

Over half the participants (57%) reported experiencing pain from their third molar teeth on at least one occasion ( **Extended data**), while 17% reported experiencing facial swelling on at least one occasion ( **Extended data**). A smaller proportion of the cohort reported receiving surgical management (10%) or non-surgical treatment (7%) for their third molars ( **Extended data**).

Female sex was associated with greater odds for all four outcomes examined, with adjusted odds ratios (OR) between OR 1.54 (95% confidence interval (CI) 1.17, 2.06) and OR 2.30 (95%CI 1.62, 3.35) for these outcomes ( Table 2). People who reported dental anxiety had greater odds for extraction (adjusted OR= 1.70; 95%CI 1.05, 2.66) although with wide confidence intervals. Patients with previously restored teeth had greater odds of having had at least one third molar extracted, compared to those with no previous restorations. This association was stronger for participants with a greater number of previous restorations: 5-9 previously restored teeth (adjusted OR= 1.79; 95% CI 1.25, 2.52) and > 9 previously restored teeth (adjusted OR= 2.73; 95%CI 1.56, 4.58) ( Table 2).

**Table 2.** Logistic regression models for host and environmental risk factors on third molar symptoms and treatment outcomes (OR, Odds Ratio; CI, Confidence Intervals; Ref, Reference; IMD, index of multiple deprivation, *adjusted for age, sex and IMD quantile).

	Swelling		Pain		Non-surgical management		Surgical management	
	Crude	Adjusted *	Crude	Adjusted	Crude	Adjusted	Crude	Adjusted
**Exposure**	OR (95% CI)	OR (95% CI)	OR (95% CI)	OR (95% CI)	OR (95% CI)	OR (95% CI)	OR (95% CI)	OR (95% CI)
**Sex**	P = 3.1x10 ^-12^	P = 1.86 x10 ^-8^	P=0.44	P=0.44	P=0.79	P=0.73	P=0.24	P=0.24
**Female**	2.13 (1.73, 2.65)	1.97 (1.56, 2.51)	2.13(1.85,2.47)	1.96(1.67,2.30)	2.33(1.70,3.26)	2.30(1.62,3.35)	1.42(1.12,1.82)	1.54(1.17,2.06)
**IMD**	P = 0.58	P = 0.68	P=0.78	P=0.92	P=0.72	P=0.76	P=0.16	P=0.13
**IMD 1**	Ref	Ref	Ref	Ref	Ref	Ref	Ref	Ref
**IMD 2**	1.06 (0.82, 1.37)	1.04 (0.80, 1.35)	1.01(0.84,1.23)	1.00(0.82,1.2)	1.21(0.85,1.75)	1.20(0.84,1.74)	0.67(0.48,0.93)	0.66(0.47,0.92)
**IMD 3**	1.21 (0.91, 1.61)	1.18 (0.89, 1.58)	0.95(0.77,1.18)	0.93(0.74,1.20)	1.13(0.74,1.70)	1.12(0.73,1.69)	1.00(0.70,1.40)	0.99(0.70,1.39)
**IMD 4**	1.00 (0.70, 1.39)	0.94 (0.66, 1.32)	1.04(0.81,1.33)	1.00(0.77,1.28)	0.94(0.56,1.53)	0.90(0.53,1.47)	0.87(0.57,1.30)	0.86(0.56,1.29)
**IMD 5**	1.28 (0.84, 1.91)	1.18 (0.77, 1.77)	1.20(0.87,1.67	1.09(0.78,1.52)	1.31(0.71,2.29)	1.20(0.65,2.10)	0.80(0.45,1.40)	0.77(0.43,1.29)
**Dental attendance**	P = 7.00 x10 ^-5^	P = 5.00 x10 ^-4^	P=2.03x10 ^-7^	P=1.02x10 ^-6^	P=0.0005	P=0.009	P=1.27x10 ^-9^	P=1.98x10 ^-6^
**Regular attendance**	0.58 (0.44, 0.75)	0.57(0.41,0.78)	0.63(0.52,0.75)	0.60(0.49,0.74)	0.46(0.29,0.70)	0.52(0.31,0.83)	0.22(0.13,0.35)	0.28(0.16,0.45)
**Dental anxiety**	P = 0.09	P = 0.29	P=0.003	P=0.32	P=0.32	P=0.83	P=0.002	P=0.02
** Dental anxiety**	1.38 (0.94, 2.00)	1.24 (0.82, 1.90)	1.63(1.20,2.26)	1.45(1.03,1.00)	1.33(0.73,2.26)	1.07(0.53,1.92)	1.91(1.25,2.87)	1.70(1.05,2.66)
**Filled teeth**	P = 0.34	P = 0.04	P=0.02	P=0.10	P=0.78	P=0.96	P=4.4x10 ^-5^	P=0.00064
**No filled teeth**	Ref	Ref	Ref	Ref	Ref	Ref	Ref	Ref
**Filled teeth 1–4**	1.06 (0.88, 1.28)	1.01 (0.82, 1.26)	1.14(0.99,1.31)	1.09(0.92,1.25)	1.13(0.87,1.48)	1.07(0.79,1.46)	0.96(0.75,1.21)	1.05(0.80,1.38)
**Filled teeth 5–9**	1.33 (0.97, 1.82)	1.27 (0.86, 1.26)	1.44(1.12,1.87)	1.45(1.08,1.96)	0.94(0.55,1.52)	0.99(0.55,1.69)	1.79(1.25,2.52)	1.98(1.30,2.95)
**Filled teeth >9**	1.21 (0.64, 2.12)	1.07 (0.50, 2.10)	1.19(0.75,1.92)	1.00(0.57,1.73)	1.08(0.41,2.34)	1.13(0.39,2.65)	2.73(1.56,4.58)	2.78(1.41,5.14)
**Previous extractions** ** due to decay**	P = 0.75	P = 0.70	P=0.75	P=0.45	P=0.70	P=0.70	P=0.46	P=0.30
** 0**	Ref	Ref	Ref	Ref	Ref	Ref	Ref	Ref
**1–3**	0.59 (0.32, 1.01)	0.54 (0.27, 0.96)	1.00(0.70,1.44)	0.96(0.66,1.42)	0.77(0.32,1.60)	0.74(0.28,1.60)	0.75(0.37,1.36)	0.72(0.33,1.40)
**>=4**	0.74 (0.35, 1.39)	0.59 (0.26, 1.19)	0.84(0.54,1.33)	0.73(0.45,1.20)	0.75(0.22,1.86)	0.78(0.23,2.1.95)	1.30(0.64,2.41)	1.52(0.74,2.86)
**Sugar Intake**	P = 0.20	P = 0.53	P=0.04	P=0.32	P=0.32	P=0.52	P=0.09	P=0.09
**Per 100 g**	0.99 (0.96, 1.00)	1.01 (0.98, 1.03)	0.98(0.97,1.00	0.99(0.97,1.00)	0.98(0.951,1.01)	1.01(0.97,1.04)	1.02(0.99,1.04)	1.02(1.00,1.06)

Heritability analysis yielded imprecise estimates, likely reflecting the low statistical power of this analysis in the available sample size. For pain, the estimated heritability was 0.17 (Standard Error 0.17), while the remaining traits had points estimates near zero.

## Discussion

This study aimed to describe the prevalence of third molar symptoms in a birth cohort study and describe host and environmental risk factors for developing symptomatic third molar teeth or requiring treatment, with the assumption that self-reported pain, swelling and treatment for third molar teeth would serve as proxies for underlying pathology such as caries or pericoronitis. The main findings are that third molar problems are common in young adults, and that female sex was strongly associated with self-reported third molar problems and self-reported treatment. In this group of young adults, pain associated with third molar teeth affected a much higher proportion of people than those who received treatment for third molar teeth, suggesting that there may be a large burden of sub-clinical third molar problems in this age group.

In this study, female participants had greater odds for receiving surgical and non-surgical treatment than male participants. In part, this may reflect differences in health-seeking behaviour. It is reported that women are more active in seeking help with dental problems than men ^31, 32^. This mechanism, however, would not explain the finding that female participants had greater odds of reporting pain and swelling. Other possible explanations include differences in the perception or recollection of pain, differences in the mechanics of tooth eruption (for example related to the smaller size of the female mandible ^33^, or sex-related differences in the chronology of tooth eruption). If present, a biomechanical reason for sex differences in third molar symptoms might suggest a need for sex-specific protocols in clinical management. The interplay between sex, third molar biomechanics and health-seeking behaviour cannot be fully explored in this study but is suggested as a topic for future research.

In this study irregular dental attenders had lower odds of reporting either pain or swelling from their third molars and had lower odds of receiving treatment. Interpretation of this finding is complex. While historically it was believed that prevention at regular dental attendance should associate with lower levels of dental pathology ^34^, there is now a drive in the UK for targeted recall intervals where patients deemed at highest risk of dental problems are seen most frequently, and patients with acute dental problems may attend more frequently for management of those problems. This complicates interpretation in the context of an observational study. There is a disparity between the number of participants reporting pain and those that receive any form of treatment suggesting most cases go untreated. Other UK studies have demonstrated the impact of NICE guidelines on increasing the modal age of patients receiving third molar treatment, from 26 to 29 years, which may add to the complexity of surgery and risk of complications ^35^.

In this study, participants who reported having filled teeth had greater odds of reporting third molar removal and there were directionally consistent but weak associations with third molar swelling and pain. This might reflect shared risk factors for dental caries and symptomatic third molars, for example poor oral hygiene is an established risk factor for both caries ^36^ and pericoronitis ^37^. Associations between previous dental extractions due to decay and third molar symptoms or treatment were imprecisely estimated, probably reflecting the small number of people with previous dental extractions in this cohort.

In this study IMD was not strongly associated with third molar pathology, which is in keeping with another UK third molar study ^38^, while previous publications in the same cohort show strong associations between socio-demographic variables and caries ^39, 40^. This suggests deprivation is less strongly associated with third molar symptoms than other dental diseases. We note however that power to detect an association may have been limited as the ALSPAC study cohort who were still active at the time of this questionnaire was biased towards people from less deprived backgrounds, and the deprivation scores were obtained some years before information on third molar problems. In addition, there was relatively little variance in deprivation since all the participants were originally recruited from three District Health Authorities (Southmead, Frenchay and Bristol and Weston). Thus, the quintiles of deprivation scores (assigned within the study population) do not represent the full range of deprivation seen in the UK.

Sugar intake was not associated with self-reported third molar problems or treatment in this study. This may suggest the third molar problems experienced by participants in this study are not due to caries (where sugar is a risk factor ^41^), or might reflect a limitation of the available measurement, as changes in dietary habit between completing the diet diaries at age 13 and participating in the oral health questionnaire are not captured

Host genetic factors are known to influence dento-maxillofacial morphology ^42, 43^ and govern events leading to tooth eruption. It seems plausible that host genetic factors could therefore predispose to unfavorable third molar position, morphology or available space for eruption, and could therefore be risk factors for third molar symptoms ^44^. In this study, heritability estimates using the GREML method produced wide confidence intervals. While there was weak evidence for a genetic contribution to third molar pain, larger sample sizes or other designs such as twin-based studies ^45, 46^ will be required to confirm this.

This study has the advantage of using an unascertained population so includes those who do not, or are unable to, access dental care. This should give a more representative estimate of prevalence than studies in clinical settings such as oral surgery or primary care units. While using a population-based rather than clinical design has natural advantages, it also has the disadvantage that the data were self-reported and will include both over- and under-reporting of outcomes. To try and reduce error from recall bias, data was collected at age 23, which is likely to be near the peak age for wisdom tooth problems ^47^, however a natural limitation of this is that the results represent a snapshot of third molars at one point in time and we cannot comment on associations at other points in the life course. To minimize error, the question stems needed to be simple, and this means the questions did not attempt to distinguish between different types of non-surgical treatment such as analgesic advice, mechanical cleaning of the operculum or removal of the operculum. It is not possible to comment on the risk factors for different types of non-surgical treatment or make any comments of what forms of treatment are more common in particular patient groups. In general, the risk factor profiles were similar for pain, swelling, surgical and non-surgical treatment, suggesting the available questions acted as proxies for a similar underlying condition, and detailed dissection of any one of these questions may not change the overall interpretation of results.

In summary, the study highlighted that third molar problems are common in young adults. The risk factors for third molar symptoms appear different from the risk factors for caries, given that the expected risk factors for caries such as socioeconomic status, sugar intake and irregular attendance were not strongly associated with third molar symptoms. By contrast, female sex was strongly associated with both self-reported third molar symptoms and self-reported treatment. It may be useful to investigate sex differences in third molar biomechanics and care-seeking behaviour to understand whether sex-specific third molar protocols would be useful in clinical practice. It is also important to note that, whilst these associations have been noted at age 23 they may not be consistent over an individual's life course and may change most individuals over or under the age of 23.

## Data Availability

ALSPAC data access is through a system of managed open access. The steps below highlight how to apply for access to the data included in this research article and all other ALSPAC data. The datasets presented in this article are linked to ALSPAC project number B3482, please quote this project number during your application. The ALSPAC variable codes highlighted in the dataset descriptions can be used to specify required variables. Please read the ALSPAC access policy which describes the process of accessing the data and samples in detail, and outlines the costs associated with doing so. You may also find it useful to browse our fully searchable research proposals database, which lists all research projects that have been approved since April 2011. Please submit your research proposal for consideration by the ALSPAC Executive Committee. You will receive a response within 10 working days to advise you whether your proposal has been approved. If you have any questions about accessing data, please email alspac-data@bristol.ac.uk. The study website also contains details of all the data that is available through a fully searchable data dictionary. Extended data are available at https://doi.org/10.6084/m9.figshare.19188224.v1

## References

[1] Swift JQ Nelson WJ : The Nature of Third Molars: Are Third Molars Different than Other Teeth? *Atlas Oral Maxillofac Surg Clin North Am.* 2012;20(2):159–62. 10.1016/j.cxom.2012.07.003 23021392

[2] Singh N Chaudhari S Chaudhari R : A radiographic survey of agenesis of the third molar: A panoramic study. *J Forensic Dent Sci.* 2017;9(3):130–134. 29657489 10.4103/jfo.jfds_59_16 PMC5887635

[3] McArdle LW Jones J McDonald F : Characteristics of disease related to mesio-angular mandibular third molar teeth. *Br J Oral Maxillofac Surg.* 2019;57(4):306–11. 10.1016/j.bjoms.2019.02.002 30952374

[4] Guerisoli DM de Souza RA de Sousa Neto MD : External and internal anatomy of third molars. *Braz Dent J.* 1998;9(2):91–4. 10219121

[5] Celikoglu M Miloglu O Kazanci F : Frequency of agenesis, impaction, angulation, and related pathologic changes of third molar teeth in orthodontic patients. *J Oral Maxillofac Surg.* 2010;68(5):990–5. 10.1016/j.joms.2009.07.063 20096980

[6] Royal College of Surgeons Faculty of Dental Surgery: Parameters of care for patients undergoing mandibular third molar surgery.2020. Reference Source

[7] Friedman JW : The prophylactic extraction of third molars: a public health hazard. *Am J Public Health.* 2007;97(9):1554–9. 10.2105/AJPH.2006.100271 17666691 PMC1963310

[8] National Institute of Care Excellence: Guidance on the Extraction of Wisdom Teeth.2000. Reference Source

[9] Mansoor J Jowett A Coulthard P : NICE or not so NICE? *Br Dent J.* 2013;215(5):209–12. 10.1038/sj.bdj.2013.832 24029981

[10] Renton T Al-Haboubi M Pau A : What has been the United Kingdom's experience with retention of third molars? *J Oral Maxillofac Surg.* 2012;70(9 Suppl 1):S48–57. 10.1016/j.joms.2012.04.040 22762969

[11] McArdle LW McDonald F Jones J : Distal cervical caries in the mandibular second molar: an indication for the prophylactic removal of third molar teeth? Update. *Br J Oral Maxillofac Surg.* 2014;52(2):185–9. 10.1016/j.bjoms.2013.11.007 24314915

[12] Marciani RD : Is there pathology associated with asymptomatic third molars? *J Oral Maxillofac Surg.* 2012;70(9 Suppl 1):S15–9. 10.1016/j.joms.2012.04.025 22717377

[13] Magraw CB Golden B Phillips C : Pain with pericoronitis affects quality of life. *J Oral Maxillofac Surg.* 2015;73(1):7–12. 10.1016/j.joms.2014.06.458 25262404

[14] McGrath C Comfort MB Lo EC : Can third molar surgery improve quality of life? A 6-month cohort study. *J Oral Maxillofac Surg.* 2003;61(7):759–63; discussion 64–5. 10.1016/s0278-2391(03)00150-2 12856246

[15] Ghaeminia H Nienhuijs ME Toedtling V : Surgical removal versus retention for the management of asymptomatic disease-free impacted wisdom teeth. *Cochrane Database Syst Rev.* 2020;5(5): CD003879. 10.1002/14651858.CD003879.pub5 32368796 PMC7199383

[16] Briguglio F Zenobio EG Isola G : Complications in surgical removal of impacted mandibular third molars in relation to flap design: clinical and statistical evaluations. *Quintessence Int.* 2011;42(6):445–53. 21519580

[17] Isola G Matarese M Ramaglia L : Efficacy of a drug composed of herbal extracts on postoperative discomfort after surgical removal of impacted mandibular third molar: a randomized, triple-blind, controlled clinical trial. *Clin Oral Investig.* 2019;23(5):2443–2453. 10.1007/s00784-018-2690-9 30311061

[18] Chuang SK Perrott DH Susarla SM : Age as a risk factor for third molar surgery complications. *J Oral Maxillofac Surg.* 2007;65(9):1685–92. 10.1016/j.joms.2007.04.019 17719384

[19] Hounsome J Pilkington G Mahon J : Prophylactic removal of impacted mandibular third molars: a systematic review and economic evaluation. *Health Technol Assess.* 2020;24(30):1–116. 10.3310/hta24300 32589125 PMC7336222

[20] Donaldson AN Everitt B Newton T : The effects of social class and dental attendance on oral health. *J Dent Res.* 2008;87(1):60–4. 10.1177/154405910808700110 18096895

[21] Fernando S Tadakamadla SK Bakr M : Indicators of Risk for Dental Caries in Children: A Holistic Approach. *JDR Clin Trans Res.* 2019;4(4):333–41. 10.1177/2380084419834236 31039050

[22] Boyd A Golding J Macleod J : Cohort Profile: the 'children of the 90s'--the index offspring of the Avon Longitudinal Study of Parents and Children. *Int J Epidemiol.* 2013;42(1):111–27. 10.1093/ije/dys064 22507743 PMC3600618

[23] Fraser A Macdonald-Wallis C Tilling K : Cohort Profile: the Avon Longitudinal Study of Parents and Children: ALSPAC mothers cohort. *Int J Epidemiol.* 2013;42(1):97–110. 10.1093/ije/dys066 22507742 PMC3600619

[24] Northstone K Lewcock M Groom A : The Avon Longitudinal Study of Parents and Children (ALSPAC): an update on the enrolled sample of index children in 2019 [version 1; peer review: 2 approved]. *Wellcome Open Res.* 2019;4:51. 10.12688/wellcomeopenres.15132.1 31020050 PMC6464058

[25] Harris PA Taylor R Thielke R : Research electronic data capture (REDCap)--a metadata-driven methodology and workflow process for providing translational research informatics support. *J Biomed Inform.* 2009;42(2):377–81. 10.1016/j.jbi.2008.08.010 18929686 PMC2700030

[26] ALSPAC. Reference Source

[27] Yang J Lee SH Goddard ME : GCTA: a tool for genome-wide complex trait analysis. *Am J Hum Genet.* 2011;88(1):76–82. 10.1016/j.ajhg.2010.11.011 21167468 PMC3014363

[28] Lee SH Wray NR Goddard ME : Estimating missing heritability for disease from genome-wide association studies. *Am J Hum Genet.* 2011;88(3):294–305. 10.1016/j.ajhg.2011.02.002 21376301 PMC3059431

[29] Taylor AE Jones HJ Sallis H : Exploring the association of genetic factors with participation in the Avon Longitudinal Study of Parents and Children. *Int J Epidemiol.* 2018;47(4):1207–1216. 10.1093/ije/dyy060 29800128 PMC6124613

[30] Delaneau O Marchini J Zagury JF : A linear complexity phasing method for thousands of genomes. *Nat Methods.* 2011;9(2):179–81. 10.1038/nmeth.1785 22138821

[31] Slack-Smith LM Mills CR Bulsara MK : Demographic, health and lifestyle factors associated with dental service attendance by young adults. *Aust Dent J.* 2007;52(3):205–9. 10.1111/j.1834-7819.2007.tb00490.x 17969289

[32] Furuta M Ekuni D Irie K : Sex differences in gingivitis relate to interaction of oral health behaviors in young people. *J Periodontol.* 2011;82(4):558–65. 10.1902/jop.2010.100444 20936916

[33] Fan Y Penington A Kilpatrick N : Quantification of mandibular sexual dimorphism during adolescence. *J Anat.* 2019;234(5):709–717. 10.1111/joa.12949 30834524 PMC6481415

[34] Richards W Ameen J : The impact of attendance patterns on oral health in a general dental practice. *Br Dent J.* 2002;193(12):697–702; discussion 695. 10.1038/sj.bdj.4801664 12529726

[35] McArdle LW Renton T : The effects of NICE guidelines on the management of third molar teeth. *Br Dent J.* 2012;213(5):E8. 10.1038/sj.bdj.2012.780 22955790

[36] Selwitz RH Ismail AI Pitts NB : Dental caries. *Lancet.* 2007;369(9555):51–9. 10.1016/S0140-6736(07)60031-2 17208642

[37] Moloney J Stassen LF : Pericoronitis: treatment and a clinical dilemma. *J Ir Dent Assoc.* 2009;55(4):190–2. 19753908

[38] Fernandes MJ Ogden GR Pitts NB : Incidence of symptoms in previously symptom-free impacted lower third molars assessed in general dental practice. *Br Dent J.* 2009;207(5):E10; discussion 218–9. 10.1038/sj.bdj.2009.804 19730432

[39] Broomhead T Baker SR Jones K : What are the most accurate predictors of caries in children aged 5 years in the UK? *Community Dent Health.* 2014;31(2):111–6. 25055609

[40] Weston-Price S Copley V Smith H : A multi-variable analysis of four factors affecting caries levels among five-year-old children; deprivation, ethnicity, exposure to fluoridated water and geographic region. *Community Dent Health.* 2018;35(4):217–22. 10.1922/CDH_4383Weston-Price06 30188616

[41] Hong J Whelton H Douglas G : Consumption frequency of added sugars and UK children's dental caries. *Community Dent Oral Epidemiol.* 2018;46(5):457–464. 10.1111/cdoe.12413 30125961

[42] Townsend G Hughes T Luciano M : Genetic and environmental influences on human dental variation: a critical evaluation of studies involving twins. *Arch Oral Biol.* 2009;54 Suppl 1(Suppl 1):S45–51. 10.1016/j.archoralbio.2008.06.009 18715551 PMC2981882

[43] Švalkauskienė V Šmigelskas K Šalomskienė L : Heritability estimates of dental arch parameters in Lithuanian twins. *Stomatologija.* 2015;17(1):3–8. 26183851

[44] Carter K Worthington S : Morphologic and Demographic Predictors of Third Molar Agenesis: A Systematic Review and Meta-analysis. *J Dent Res.* 2015;94(7):886–94. 10.1177/0022034515581644 25883107

[45] Boomsma D Busjahn A Peltonen L : Classical twin studies and beyond. *Nat Rev Genet.* 2002;3(11):872–82. 10.1038/nrg932 12415317

[46] Yang J Zeng J Goddard ME : Concepts, estimation and interpretation of SNP-based heritability. *Nat Genet.* 2017;49(9):1304–1310. 10.1038/ng.3941 28854176

[47] Renton T Wilson NH : Problems with erupting wisdom teeth: signs, symptoms, and management. *Br J Gen Pract.* 2016;66(649):e606–8. 10.3399/bjgp16X686509 27481985 PMC4979926

