# Supplementary material for: An observational analysis of risk factors associated with symptomatic third molar teeth
Source: Wellcome Open Res. 2023 Jul 17;7:71. Originally published 2022 Feb 28. [Version 2] doi: 10.12688/wellcomeopenres.17673.2 (PMC10422052; doi:10.12688/wellcomeopenres.17673.2)
Supplement: Peer review history

# Peer review: Reviewer response for version 2

**DOI:** 10.21956/wellcomeopenres.21726.r63030

**Published:** 2023-08-11

**Authors:** Gaetano Isola

**Affiliations:** 1 Department of General Surgery and Surgical-Medical Specialties, School of Dentistry, University of Catania, Catania, Italy

The authors have well addressed all of the issues raised by the reviewer. No further comments are needed.

Is the work clearly and accurately presented and does it cite the current literature?

Partly

If applicable, is the statistical analysis and its interpretation appropriate?

Yes

Are all the source data underlying the results available to ensure full reproducibility?

No

Is the study design appropriate and is the work technically sound?

Yes

Are the conclusions drawn adequately supported by the results?

Partly

Are sufficient details of methods and analysis provided to allow replication by others?

Partly

Reviewer Expertise:

Dentistry, oral surgery

I confirm that I have read this submission and believe that I have an appropriate level of expertise to confirm that it is of an acceptable scientific standard.

# Peer review: Reviewer response for version 1

**DOI:** 10.21956/wellcomeopenres.19551.r53362

**Published:** 2023-02-27

**Authors:** Gaetano Isola

**Affiliations:** 1 Department of General Surgery and Surgical-Medical Specialties, School of Dentistry, University of Catania, Catania, Italy

In the manuscript entitled: "An observational analysis of risk factors associated with symptomatic third molar teeth" the authors examined the prevalence of symptomatic third molar teeth and identify factors which predispose to third molar symptoms in a birth cohort.

 The authors found that females had greater odds than males of reporting swelling (adjusted odds ratio (OR) 1.97; 95% confidence interval (CI) 1.56, 2.51), pain (adjusted OR=1.96; 95%CI 1.56, 2.51) and receiving both non-surgical and surgical treatment (adjusted OR=2.30; 95%CI 1.62, 3.35, adjusted OR=1.54; 95%CI 1.17, 2.06 respectively). Participants with previously filled teeth had greater odds of third molar extraction. There were no strong associations between index of multiple deprivation (IMD) score or sugar intake and the third molar outcomes. There was weak evidence for a genetic contribution to third molar pain.

 The authors concluded that symptomatic third molars are common in this age group, with over half of the participants reporting pain or other symptoms. Female participants had greater odds for third molar pain, swelling and treatment.

**Major comments:**

 In general, the idea and innovation of this study regards the analysis of mediators during periodontitis is interesting and novel because because the role these aspects have in medicine are validated but further studies on this topic could be an innovative issue in this field and could be open a creative matter of debate in literature by adding new information. Moreover, there are few reports in the literature that studied this interesting topic with this kind of study design.

 The study was well conducted by the authors; However, there are some concerns to revise that are described below.

- The introduction section resumes the existing knowledge regarding the important factors linked with third molar and related outcomes. However, as the importance of the topic, the reviewer strongly recommends, before a further re-evaluation of the manuscript, to update the literature, particularly through reading, discussing, and citing the following recent interesting articles, which will help the authors to better introduce and discuss the role of third molar flap and natural agents. 1) doi: 10.1007/s00784-018-2690-9. PMID: 30311061 2) PMID: 21519580
- The authors should better specify, at the end of the introduction section, the rational of the study and the aim of the study. In the central section, they should better clarify inclusions and exclusions criteria of the selected sample.
- The discussion section appears well organized with the relevant papers that support the conclusions, the authors should better discuss the relationship regarding the role exerted surgical flaps and natural agents in the management of the third molar surgery. Please specify the role of dental loupes in the diagnosis of periodontal depth recording. The conclusion should reinforce in light of the discussions.

In conclusion, I am sure that the authors will achieve very nice results with their adopted protocol. However, this study, in my view does not in its current form satisfy a very high scientific requirement for indexing and request a revision before a further re-evaluation of the manuscript.

**Minor Comments:**

**Abstract:**

- Better formulate the abstract section by better describing the aim of the study

**Introduction:**

- Please refer to major comments

**Discussion**

- Please add a specific sentence that clarifies the results obtained in the first part of the discussion

Is the work clearly and accurately presented and does it cite the current literature?

Partly

If applicable, is the statistical analysis and its interpretation appropriate?

Yes

Are all the source data underlying the results available to ensure full reproducibility?

No

Is the study design appropriate and is the work technically sound?

Yes

Are the conclusions drawn adequately supported by the results?

Partly

Are sufficient details of methods and analysis provided to allow replication by others?

Partly

Reviewer Expertise:

Dentistry, oral surgery

I confirm that I have read this submission and believe that I have an appropriate level of expertise to confirm that it is of an acceptable scientific standard, however I have significant reservations, as outlined above.

## References

[1] : Efficacy of a drug composed of herbal extracts on postoperative discomfort after surgical removal of impacted mandibular third molar: a randomized, triple-blind, controlled clinical trial. *Clin Oral Investig* .2019;23(5) : 10.1007/s00784-018-2690-9 2443-2453 10.1007/s00784-018-2690-9 30311061

[2] : Complications in surgical removal of impacted mandibular third molars in relation to flap design: clinical and statistical evaluations. *Quintessence Int* .2011;42(6) :445-53 21519580

# Peer review: Reviewer response for version 1

**DOI:** 10.21956/wellcomeopenres.19551.r52215

**Published:** 2022-09-08

**Authors:** James Allison

**Affiliations:** 1 School of Dental Sciences, Faculty of Medical Sciences, Newcastle University, Newcastle upon Tyne, UK

The authors present a well described and well conducted study of the prevalence of symptomatic third molar teeth and associated interventions in an established longitudinal population cohort.

 The study found that those with greater experience of dental treatment or disease, were more likely to experience third molar symptoms and interventions. Interestingly, however, there was no association between the outcomes and deprivation and sugar intake which are both predictors of dental decay.

 I have little to add to the manuscript, as it is well reported and the authors have appropriately interpreted the results. Although the authors mention that collecting diet and IMD data at a different time to outcome data my have affected the results, I would suggest that this is a fair limitation to their conclusion that neither variable affects the outcomes. The authors could make this limitation a little more prominent in the discussion, as it may be there was truly an association that the study design could not resolve. Similarly, it should be more prominent that although the reported associations exist at age 23, this may not remain the case further into the life course. It was also unclear when exactly the genotyping was performed, and what samples were obtained (e.g., saliva, buccal swabs); this should be included under the " *Data collection*" heading.

 Minor points are that the figures in the right column of *Table 1* are misaligned with their respective headings in the left column, and that the reporting of *p *values in *Table 2 *is inconsistent; it might be easier to use conventional notation (i.e. 0.003) for values larger than 0.001, and scientific notation (i.e. 1 x 10 ^-4^) for values smaller than this.

Is the work clearly and accurately presented and does it cite the current literature?

Yes

If applicable, is the statistical analysis and its interpretation appropriate?

Yes

Are all the source data underlying the results available to ensure full reproducibility?

Yes

Is the study design appropriate and is the work technically sound?

Yes

Are the conclusions drawn adequately supported by the results?

Yes

Are sufficient details of methods and analysis provided to allow replication by others?

Yes

Reviewer Expertise:

Oral surgery; orofacial pain; aerobiology;

I confirm that I have read this submission and believe that I have an appropriate level of expertise to confirm that it is of an acceptable scientific standard.
