# Supplementary material for: An observational analysis of risk factors associated with symptomatic third molar teeth
Source: Wellcome Open Res. 2022 Feb 28;7:71. [Version 1] doi: 10.12688/wellcomeopenres.17673.1 (PMC10422052; doi:10.12688/wellcomeopenres.17673.1)
Supplement: Peer review history

# Reviewer report: Reviewer response for version 1

**DOI:** 10.21956/wellcomeopenres.19551.r50652

**Published:** 2024-05-14

**Authors:** Edmund Bailey

**Affiliations:** 1 Institute of Dentistry, Barts and The London School of Medicine and Dentistry, Queen Mary University of London, London, UK

Thank you for asking me to review this study. This is a very large data set and has the potential to be used to plan services. I am not aware of any similar data sets on this scale. I would like to statistics to be clarified, but this seems to show some interesting findings in relation to gender, sugar intake and genetics. This may change the way that risk factors are viewed for third molar surgery. I support the indexing of this paper in its current form.

Is the work clearly and accurately presented and does it cite the current literature?

Yes

If applicable, is the statistical analysis and its interpretation appropriate?

I cannot comment. A qualified statistician is required.

Are all the source data underlying the results available to ensure full reproducibility?

Yes

Is the study design appropriate and is the work technically sound?

Yes

Are the conclusions drawn adequately supported by the results?

Yes

Are sufficient details of methods and analysis provided to allow replication by others?

Yes

Reviewer Expertise:

Third molar surgery and evidence based techniques. Education and scholarship.

I confirm that I have read this submission and believe that I have an appropriate level of expertise to confirm that it is of an acceptable scientific standard.
